# Aged Lens Epithelial Cells Suppress Proliferation and Epithelial–Mesenchymal Transition-Relevance for Posterior Capsule Opacification

**DOI:** 10.3390/cells11132001

**Published:** 2022-06-22

**Authors:** Zongbo Wei, Pasley Gordon, Caili Hao, Jingru Huangfu, Emily Fan, Xiang Zhang, Hong Yan, Xingjun Fan

**Affiliations:** 1 Department of Cellular Biology and Anatomy, Medical College of Georgia at Augusta University, 1460 Laney Walker Blvd., CB Building, Room CB1119, Augusta, GA 30912, USA; zowei@augusta.edu (Z.W.); chao@augusta.edu (C.H.); jhuangfu@augusta.edu (J.H.); 2 Department of Ophthalmology, Medical College of Georgia at Augusta University, Augusta, GA 30912, USA; pagordon@augusta.edu; 3 Lakeside High School at Columbia County, Evans, GA 30809, USA; emilyfan3140@gmail.com; 4 Genomics, Epigenomics and Sequencing Core, Department of Environmental and Public Health Sciences, University of Cincinnati, Cincinnati, OH 45221, USA; zhanx5@ucmail.uc.edu; 5 Xi’an Fourth Hospital, Xi’an Jiaotong University, Xi’an 710049, China; yan2128ts@med.nwu.edu.cn

**Keywords:** cataracts, posterior capsule opacification, PCO, epithelial–mesenchymal transition, EMT, aging, cataract surgery, lens

## Abstract

Posterior capsule opacification (PCO) is a frequent complication after cataract surgery, and advanced PCO requires YAG laser (Nd: YAG) capsulotomy, which often gives rise to more complications. Lens epithelial cell (LEC) proliferation and transformation (i.e., epithelial–mesenchymal transition (EMT)) are two critical elements in PCO initiation and progression pathogenesis. While PCO marginally impacts aged cataract surgery patients, PCO incidences are exceptionally high in infants and children undergoing cataract surgery. The gene expression of lens epithelial cell aging and its role in the discrepancy of PCO prevalence between young and older people have not been fully studied. Here, we conducted a comprehensive differentially expressed gene (DEG) analysis of a cell aging model by comparing the early and late passage FHL124 lens epithelial cells (LECs). *In vitro*, TGFβ2, cell treatment, and *in vivo* mouse cataract surgical models were used to validate our findings. We found that aged LECs decelerated rates of cell proliferation accompanied by dysregulation of cellular immune response and cell stress response. Surprisingly, we found that LECs systematically downregulated epithelial–mesenchymal transition (EMT)-promoting genes. The protein expression of several EMT hallmark genes, e.g., fibronectin, αSMA, and cadherin 11, were gradually decreased during LECs aging. We then confirmed these findings *in vitro* and found that aged LECs markedly alleviated TGFβ2-mediated EMT. Importantly, we explicitly confirmed the *in vitro* findings from the *in vivo* mouse cataract surgery studies. We propose that both the high proliferation rate and EMT-enriched young LECs phenotypic characteristics contribute to unusually high PCO incidence in infants and children.

## 1. Introduction

According to the World Health Organization (WHO), at least 94 million people worldwide have cataracts, a leading cause of blindness. Most cataracts are related to aging, referred to as age-related cataracts (ANC). In addition, an estimated 200,000 children worldwide are blinded due to congenital or developmental cataracts, and an estimated 20,000 to 40,000 newborns each year carry congenital cataracts [1]. Currently, cataract surgery is the only treatment option for the disease. Approximately 4 million and 28 million cataract surgical procedures are performed annually in the US and worldwide, respectively. Despite a well-established surgical procedure, posterior capsule opacification (PCO) is a frequent complication after cataract surgery, also known as a secondary cataract. If PCO sufficiently reduces vision, patients must undergo a YAG laser (Nd: YAG) capsulotomy to remove PCO tissue and restore a clear line of sight. In addition to added healthcare costs for cataract patients, Nd: YAG capsulotomy is often associated with other complications, such as increasing transient intraocular pressure, lens subluxation and dislocation, retinal detachment and lens pitting, exacerbation of local endophthalmitis, and free-floating fragments [2].

The prevalence of PCO is closely associated with patient age, surgical techniques, the surgeon’s experience, and the materials and design of the intraocular lens (IOL). The PCO incidence rate is exceptionally high for infants and young children when the posterior capsule is retained during the surgery. Basti et al. [3] found a 43.7% PCO rate at the 11-month follow-up of 87 extracapsular cataract extraction with IOL implantation (ECCE+IOL) patients between 2 and 4 years of age. Knight-Nanan et al. [4] found a 95.8% PCO occurrence at the 2-year follow-up of 24 ECCE plus IOL implantation eyes of patients ranging from 4 weeks to 12 years old. Clinical studies found close to 100% PCO incidence rates in pediatric cataract surgeries via ECCE plus IOL implantation [5,6,7]. For this reason, primary posterior capsulotomy and anterior vitrectomy are now routine surgical strategies in pediatric cataract surgery [8].

The PCO incidence is much lower in adult cataract patients after surgery. Several large-size retrospective cohort studies have examined the prevalence of PCO while comparing the impact of IOL material and design on PCO formation. Ursell et al. [9] assessed a 3-year incidence of Nd: YAG capsulotomy and PCO among 52,162 eyes from 39,324 patients aged 65 and older with cataract surgery with IOL implantation made from different materials. The Nd: YAG capsulotomy incidence rate was between 2.4% and 10.9%, and the PCO rate was between 4.7% and 14.8%. In a separate study, a 5-year follow-up of electronic record examinations of 20,763 eyes from 16,595 patients aged 65 and older who had cataract surgery with single-piece IOL implantation revealed a 7.1–22.6% PCO incidence rate [10]. It is worth noting that elevated PCO incidence rates were found in patients younger than 60 years from a retrospective cohort study that examined the record of 10,044 eye surgeries [11].

PCO originates from residual lens epithelial cells (LECs) after cataract surgery, which will eventually deposit at the posterior capsule through survival, proliferation, migration, and transformation [12]. LEC proliferation and transformation are two critical components in PCO initiation and progression. Continuous proliferation and sometimes hyperproliferation is vital to LEC survival, and newly proliferated LECs can expand and migrate toward the posterior capsule [13,14]. LEC transformation is considered a critical process that alters the LEC phenotype by acquiring mesenchymal-like functions, a more invasive type. The epithelial–mesenchymal transition (EMT) is believed to be the primary mechanism driving LEC transformation [15,16,17].

Due to the nature of the disease, the need for cataract surgery primarily impacts either the very young or elderly population. The discrepancy in PCO incidences between young and aged people raises an essential question of how LECs respond to wound healing originating from similar surgical procedures and how LEC transformation impacts PCO progression at different ages. Currently, a commonly accepted explanation is that young lens epithelial cells are more proliferative than aged LECs since it has been well documented that cell division rates decrease with age [18,19]. However, PCO formation is widely believed to result from complex and multifactorial processes. The actual mechanisms responsible for the discrepancy in PCO prevalence between young and aged groups remain unknown. The present study discovered that aged LECs not only decelerate proliferation but also suppress epithelial–mesenchymal transition (EMT) based on a comprehensive comparative transcriptomic analysis of our established LEC aging model. We then confirmed the in vitro findings using the *in vivo* mouse cataract surgery model. 

## 2. Methods

### 2.1. Reagents

All chemicals used were of analytical reagent grade. Milli-Q water was used for the preparation of standards and reagents. DAPI (23018) was purchased from Biotium (Fremont, CA, USA). Hoechst33342 (H3570) was purchased from Thermo Fisher Scientific (Waltham, MA, USA). Antibody to fibronectin (P1H11) was obtained from the Developmental Studies Hybridoma Bank (DSHB, Iowa City, IA, USA). Fibronectin (Ab268020) and tenascin C (ab108930) antibodies were purchased from Abcam (Cambridge, MA, USA). αSMA antibody (F3777) was purchased from Millipore-Sigma (St. Louis, MO, USA). N-cadherin antibody (14215) was purchased from Cell Signaling Technology (Danvers, MA, USA). Cadherin-11 antibody (368702) was purchased from BioLegend (San Diego, CA, USA). ZO-1 (40-2200) and GAPDH (PA1-987) antibodies were purchased from Thermo Fisher Scientific. The Cell Counting Kit-8 assay kit (HY-K0301) was purchased from MedChem Express (Monmouth Junction, NJ, USA). All other chemicals were obtained from Sigma-Aldrich and Thermo Fisher Scientific.

### 2.2. Cell Culture and Lens Epithelial Cell Aging Model

The human lens epithelial cell line, FHL124 cells [20], established by Prof. John Reddan at Oakland University, was supplied by Prof. Michael Wormstone at the University of East Anglia, UK, and was grown in MEM with 5% FBS and 100 U/mL of penicillin and streptomycin at 35 °C in a humidified 5% CO_2_ incubator.

The FHL124 cell cellular aging model was established following our previous study, [21]. In brief, FHL124 cells were continuously cultured and split when cells reached 80% confluence. For early passage (young) FHL124 cells, the doubling time is around two days; for late passage (old) FHL124 cells, the doubling time is around 3–4 days.

### 2.3. Animals

All animal experiments were performed by procedures approved by the Augusta University Animal Care and Use Committee and conformed to the ARVO Statement for the Use of Animals in Ophthalmic and Vision Research. Animals were housed under a diurnal lighting condition and allowed free access to food and water. C57BL/6J mice at 2 and 20 months were purchased from the National Institute of Aging (NIA) aged rodent colonies.

### 2.4. Cell Proliferation Assay

The rate of cell proliferation was determined by colorimetric assay using the Cell Counting Kit-8 assay following the manufacturer’s instructions. In brief, 2000 cells per well were placed in a 96-well-plate, and cell division was measured every 24 h for seven days. At each time point, 10 µL of CCK-8 solution was added to each well, and cells were incubated at 35 °C for 1.5 h before recording the absorbance at 450 nm. The cell-free medium served as the blank.

### 2.5. RNAseq and Data Analysis

For the RNAseq study, we used passage 15 (P15) and 42 (P42) FHL124 cells. The next-generation deep sequencing was carried out by the Genomics, Epigenomics, and Sequencing Core at the University of Cincinnati with a similar procedure as our previous study [22]. Three biological replicates of each passage were sequenced (n = 3). The RNA quality control, polyRNA enrichment, and library preparation were described in our previous study [21]. The library was indexed and amplified under 11 PCR cycle numbers. After library Bioanalyzer QC analysis and quantification via real-time PCR (NEBNext Library Quant Kit, NEB), the proportionally pooled libraries were sequenced using a NextSeq 550 sequencer (Illumina, San Diego, CA, USA). Under the sequencing setting of single read 1 × 85 bp, about 25 million pass filter reads per sample were generated. 

The deep sequencing data were processed using Partek Flow. Unaligned reads were trimmed based on a PHRED quality score with a minimal read >25 nt. Trimmed reads were aligned to the latest human genome assembly hg19 using a STAR aligner. Aligned reads were quantitatively aligned to Ensembl Transcripts release 100. Raw counts were further filtered and normalized to transcripts per million (TPM). Deferentially expressed genes (DEGs) and pairwise comparison of p15 and p42 were extrapolated and normalized using a negative binomial model in DESeq2. The significantly DEGs between groups were expressed as fold change or log_2_ fold change with *p*-value < 0.05 and false discovery rate (FDR < 0.05). RNAseq data are deposited in the Gene Expression Omnibus (GEO) database under accession number GSE184422.

The FVB/N mouse lens epithelium deep sequencing data [23] were obtained from NCBI’s Sequence Read Archive (SRA accession number: SRP040480), CD1 mouse lens epithelium deep sequence data [24] were obtained from GEO (accession number: GSE113887), and C57BL/6 mouse epithelium deep sequence data [25] were obtained from GEO (accession number: GSE166619). All raw sequencing data with fastq format were analyzed and normalized through Partek Flow using the same calculation and normalization method as that of FHL124 cells.

### 2.6. Gene Set Enrichment Analysis (GSEA)

GSEA is a powerful gene enrichment tool developed by a joint project of UC San Diego and Broad Institute using a pre-ranked gene database based on the fold changes of all gene expression profiles in contrast to taking only significant DEGs [26]. P42 and P15 passage gene sets were analyzed for the Molecular Signature Database (MSigDB 7.4). To identify the most relevant biological processes, we only selected the nominal *p*-value below 0.05, a false discovery rate less than 15%, and a normalized enrichment score (NES) > 1.40 or <−1.30. The resulting normalized enrichment scores (NESs) were then used to create heatmaps, and enriched genes were further analyzed based on their relative expression via transcript per million (TPM).

### 2.7. TGFβ2 Treatment

Cells at different passages were seeded in 35 mm culture dishes at a density of 1.0 × 10^5^ cells/dish 16 h before treatment. Cell medium was then replaced with freshly prepared medium with or without 10 ng/mL TGFβ2. Then, 24 h after treatment, cells were washed three times with cold PBS before being harvested for immunoblot assay.

### 2.8. Cell Immunofluorescence Staining

FHL124 cells were seeded on a 35 mm round glass coverslip at a density of 3 × 10^4^/mL in a 24-well plate. After about 16 h, cells were fixed by 4% PFA at RT for 15 min. Except for ZO-1 staining, a membrane protein, fixed cells were treated with 0.5% TritonX-100 for 10 min at RT to permeabilize the cell membrane. Subsequently, coverslips were incubated with 5% normal goat serum for 30 min at RT to block non-specific background. Coverslips were incubated with appropriate primary antibodies at 4 °C overnight in a humidified chamber. Following 3 × 5 min PBST (PBS with 0.1% Tween 20) wash, coverslips were incubated with a fluorescent conjugated secondary antibody at RT for 1 h. After 3 × 5 min PBST rinse, coverslips were mounted with a mounting medium and subjected to confocal microscopy. 

### 2.9. Mouse Cataract Surgery

Mice at 2 months and 20 months of age were used in this study. Mice were anesthetized with a ketamine-xylazine before surgery. A detailed surgical procedure was described in our previous study [27]. For each mouse, left eye surgery was performed on day 1. On day 5, right eye cataract surgery was performed, and the mouse was euthanized immediately after surgery to harvest both eyes so that the right eye served as a control. Eye drops containing 0.3% of ofloxacin were applied once immediately after surgery. The harvested eyes were fixed in 4% paraformaldehyde fixative for 24 h and then subjected to paraffin embedding and sectioning.

### 2.10. Immunofluorescence Staining of Paraffin Sections

Before the antigen retrieval, sections were deparaffined and rehydrated by xylene, series gradient ethanol, and PBS. For αSMA and FN1 staining, sodium citrate antigen retrieval buffer (10 mM sodium citrate, 0.05% Tween 20, pH 6.0) was used. In contrast, TE antigen retrieval buffer (10 mM Tris base, 1 mM EDTA solution, 0.05% Tween 20, pH 9.0) was used for tenascin C staining. Sections were immersed into a proper antigen retrieval buffer and processed by the Epitope Retrieval Steamer Set (IW-1102) from IHC World (Woodstock, MD, USA) for 20 min. After 10 min cooling times at RT, slides were washed 2 × 5 min with PBS. Tissues were then permeabilized by incubating with 0.5% Triton-X-100 for 10 min at RT. Tissue slides were then blocked by 5% goat serum for 30 min at RT before being stained with an anti-αSMA antibody (1:500, F3777, Sigma-Aldrich), anti-fibronectin antibody (1:200, Ab268020, Abcam), and anti-tenascin C antibody (1:500, Ab108930, Abcam) at 4 °C overnight. The next day, sections were washed 3 × 5 min with PBST before being incubated with proper secondary antibody conjugated with fluorescent dye at RT for 1 h. After a 3 × 5 min wash with PBST, the sections were mounted with a mounting medium and ready for confocal microscopy.

### 2.11. Confocal Microscopy

All images were captured by a Leica STELLARIS confocal microscopy (Leica) and analyzed by LAS-X software.

### 2.12. Immunoblot Assay

Immunoblot assays were performed as previously described by [27]. In brief, the protein concentration from the supernatant was measured by protein bicinchoninic acid (BCA) assay (Thermo Fisher Scientific). Equal amounts of protein were subjected to appropriate SDS-PAGE gel electrophoresis and transferred to a 0.45 μm pore PVDF membrane. Detection was done using the ECL Western blotting detection system.

### 2.13. Statistics

All values are expressed as mean ± SD. In short, one-way ANOVA with Tukey’s honest post hoc analysis and 2-way ANOVA with Holm–Šídák’s multiple-comparisons test were computed using SPSS and GraphPad 9 software. Significance was considered at *p* < 0.05.

## 3. Results

### 3.1. FHL124 Is a Suitable Human Lens Epithelial Cell Line for Aging Study

**Figure 1:**
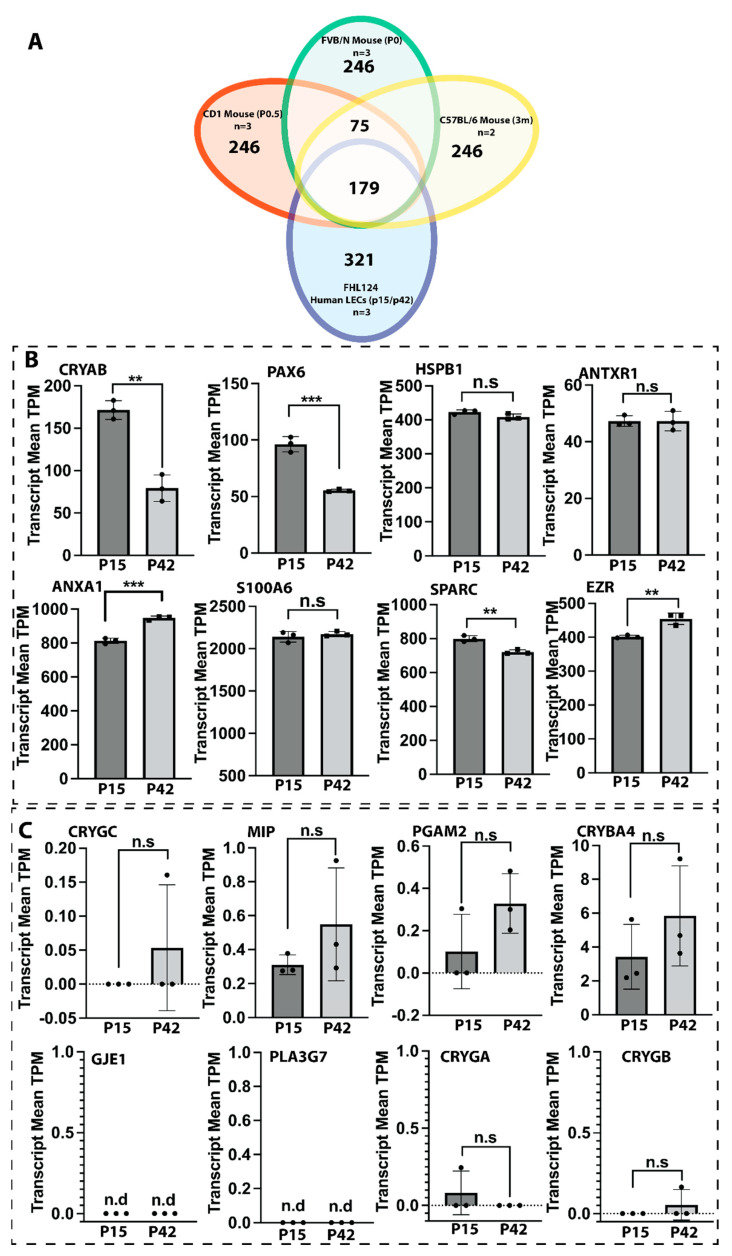
Normalized pro-LEC and pro-fiber cell gene expression in P15 and P42 FHL124 cells. (**A**) Shared and distinct top 500 most expressed genes between mouse lens epithelium and the human FHL124 LECs. (**B**) Pro-LEC gene transcript means count measured as transcript per million (TPM). (**C**) Pro-fiber gene transcript means count measured as transcript per million (TPM). Results are expressed as mean ± SD and were analyzed using Student’s t-test. Only *p* < 0.05 is considered significant. * < 0.05, ** < 0.01, *** < 0.001, **** < 0.0001; n.s, not significant; n.d, not detectable.

Aiming to understand the role of gene expression in the discrepancy of PCO incidence rates between young and older adults, we performed a comparative transcriptome analysis of FHL124 human lens epithelial cells between their early passage (P15) and late passage (P42). FHL124 cells are spontaneously grown out of human lens epithelial cell lines without artificial immortalization that share 99.5% homology with native human lens epithelium [28,29]. Continuous cell division is an ideal cellular aging model for studying LEC aging. We have found that this in vitro cellular aging model closely resembles the *in vivo* situation [21]. FHL124 cells reached their senescence around passage 55 (P55) [21], and for this study, we performed all our assays before passage 50 (P50) to avoid a complete senescence state. To further validate the FHL124 cells’ LEC characteristics at both early and late passages, we compared the top 500 most expressed genes between FHL124 cells and three strains (FVB/N, age p0 [23]; CD1, age p0.5 [24]; and C57BL/6, age 3 months [25]) of mouse lens epithelium under the same transcript normalization method. There are 245 genes shared between three strains of mouse lens epithelium. Notably, 179 over 245 genes (70.4%) also sit in the top 500 most expressed genes in FHL124 cells at P15 and P42 (Figure 1A). All the shared genes are listed in Table S1. Furthermore, we also surveyed the pro-LEC gene expression profile of P15 and P42 passage FHL124 cells. From 300 lens top-enriched genes ranked by iSyTE [30], we found that 246 genes (82%) were expressed, and 161 genes (54%) were significantly expressed (top 1000 most expressed genes) in FHL124 cells at both passage 15 and 42. Several prominent lens epithelial cell-associated genes, such as CRYAB, PAX6, HSPB1, ANTXR1, ANXA1, S100A6, SPARC, and EZR, were highly expressed in both P15 and P42 passage FHL124 cells (Figure 1B). Although we found that several of these genes had significant changes in their expression between early and late passages, their overall expression levels were abundant in both passages. Conversely, we compared several lens fiber cell markers and their expression in P15 and P42 passage FHL124 cells. As shown in Figure 1C, almost no detectable expression of CRYGC, MIP, PGAM2, CRYBA4, GJE1, PLA3G7, CRYGA, and CRYGB was found in early and late passage FHL124 cells. These results suggest that FHL124 cells closely resemble the lens epithelium and are suitable for lens biology and aging studies.

### 3.2. Aging Induces Substantial Gene Expression Changes in Lens Epithelial Cells

**Figure 2:**
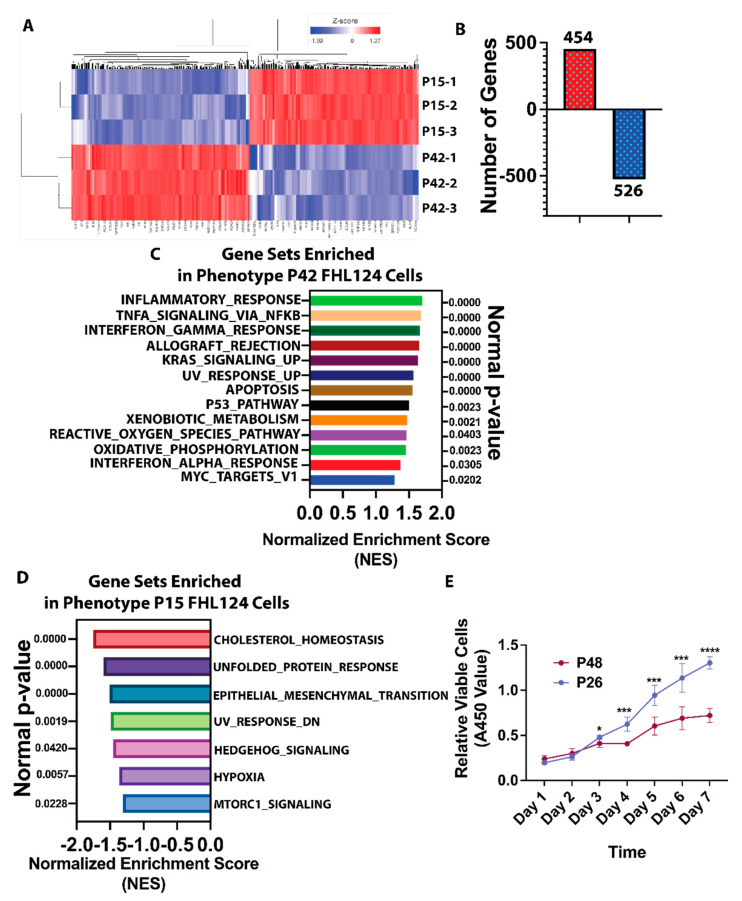
Aging alters LEC gene expression and decelerates LECs’ proliferation. Passage 15 (n = 3) and passage 42 (n = 3) FHL124 cells were used for RNAseq, and normalized transcriptomes were comparatively analyzed between P42 and P15 cells. (**A**) RNAseq heatmap showing the top 1000 genes differentially expressed in passage 42 and passage 15 FHL124 cells. (**B**) When comparing P42 to P15, differential gene expression (DGE) analysis revealed that 454 genes were upregulated, and 526 genes were downregulated more than 2-fold with both *p*-value and false discovery rate (FDR) less than 0.05. (**C**) GSEA (gene set enrichment analysis) identified 13 top-ranked pathways enriched in P42 passage FHL124 cells with a nominal *p*-value less than 0.05 and FDR below 5%. The pathways were ranked by normalized enrichment score (NES). (**D**) GSEA using MSigDB canonical pathway identified 7 top-ranked pathways enriched in P15 passage FHL124 cells with a nominal *p*-value less than 0.05 and FDR below 15%. The pathways were ranked by normalized enrichment score (NES). (**E**) Seven-day cell proliferation curve of both P26 and P48 passage FHL124 cells was assayed by CCK8 (n = 8 at each data point). Results in E are expressed as mean ± SD and were analyzed using 2-way ANOVA with Holm–Šídák’s multiple-comparisons test. Only *p* < 0.05 is considered significant. * < 0.05, ** < 0.01, *** < 0.001, **** < 0.0001.

Over 27,000 transcripts were normalized and compared between P42 and P15 (Figure 2A, heatmap; Figure S1A, volcano plot; and Figure S1B, principal component analysis (PCA)). We found that 454 differentially expressed genes (DEGs) were upregulated, and 526 were downregulated for more than 2-fold with both a *p*-value and false discovery rate (FDR) of less than 0.05 when comparing P42 with P15 passage FHL124 cells, respectively (Figure 2B). To evaluate the status of cell senescence at P42, we performed a differential expression analysis of genes that either induce (n = 133) or inhibit (n = 108) cell senescence according to the database of cell senescence genes from the human aging genomic resource (https://genomics.senescence.info, accessed on 12 October 2021). As shown in Figure S2A–E, most cell senescence induction and inhibition genes showed no significant changes in P42 compared with P15 FHL124 cells. For example, in the case of several key cell senescence-inducing genes, e.g., p53 (~1.10 up), p16 (cyclin-dependent kinase inhibitor 2A, CDKN2A, ~1.38 up), 53BP1 (tumor protein p53 binding protein 1, TP53BP1, ~1.18 dn), and laminB1 (LMNB1, ~1.14 dn), and several essential cell senescence inhibition genes, e.g., CDK1 (cyclin-dependent kinase 1, ~1.13 up), and MYC (MYC proto-oncogene, ~1.51 dn), their expressions were mildly up- or downregulated in P42 compared with P15 passage FHL124 cells. However, we did observe several cell senescence induction genes significantly downregulated in P42LECs compared with P15, such as CDKN1C (cell cycle inhibitor cyclin-dependent kinase inhibitor 1C, ~6.96 dn). In contrast, some cell senescence induction genes were significantly upregulated, such as p21 (cyclin-dependent kinase inhibitor 1A, CDKN1A, ~5.35 up). These results suggest that FHL124 cells at passage 42 have undergone age-induced transition but have yet to reach the senescence stage.

### 3.3. Aging Decelerates Proliferation in Lens Epithelial Cells

To better understand these differentially expressed genes during the cell aging process, we conducted a comprehensive pathway analysis using the MSigDB canonical pathway (gene set enrichment analysis, GSEA). As shown in Figure 2C, a 13-hallmark gene set with a nominal *p*-value below 0.05 and FDR q-value less than 5% was significantly enriched in the late passage (P42) FHL124 cells. These enriched pathways heavily pointed to a dysregulation of the immune response, such as inflammatory (n = 200), TNFα signaling via NFκB (n = 199), IFγ response (n = 198), and allograft rejection (n = 195). This was followed by cell stress response pathways, e.g., apoptosis (n = 160), xenobiotic metabolism (n = 196), the reactive oxygen species (ROS) pathway (n = 49), and oxidative phosphorylation (n = 200). In addition, upregulated KRAS signaling (n = 199) and the p53 pathway (n = 196) were significantly enriched in aged LECs. As shown in Figure 2D, a seven-hallmark gene set with a nominal *p*-value below 0.05 and a false discovery rate (FDR) q-value less than 15% was significantly enriched in the early passage (P15) FHL124 cells. These enriched pathways include upregulated cholesterol homeostasis (n = 73), unfolded protein response (UPR) (n = 107), epithelial–mesenchymal transition (EMT) (n = 196), hedgehog signaling (n = 36), hypoxia (n = 196), mTORC1 signaling (n = 196), and downregulated UV response (n = 142).

These enriched pathways suggest that young LECs maintain vital cell survival and proliferative signaling, e.g., cholesterol homeostasis, hedgehog signaling, and mTORC1 signaling. At the same time, aged LECs adapt their cellular pathways to cope with elevated stress, e.g., ROS and apoptosis. To compare the cell proliferation rate between young and aged FHL124 cells, we performed cell proliferation analysis by Cell Counting Kit-8 (CCK-8). As demonstrated in Figure 2E, the doubling time of passage 26 (P26) FHL124 cells was around two days, while the time for passage 48 (P48) FHL124 cells was around four days. These results indicate that aged lens epithelial cells significantly decelerate cell proliferation. Also, continued cell division with a doubling time of fewer than five days further confirmed that the late passage cells we used had not yet reached cell senescence.

### 3.4. Aged Lens Epithelial Cells Suppress Epithelial–Mesenchymal Transition (EMT)

**Figure 3:**
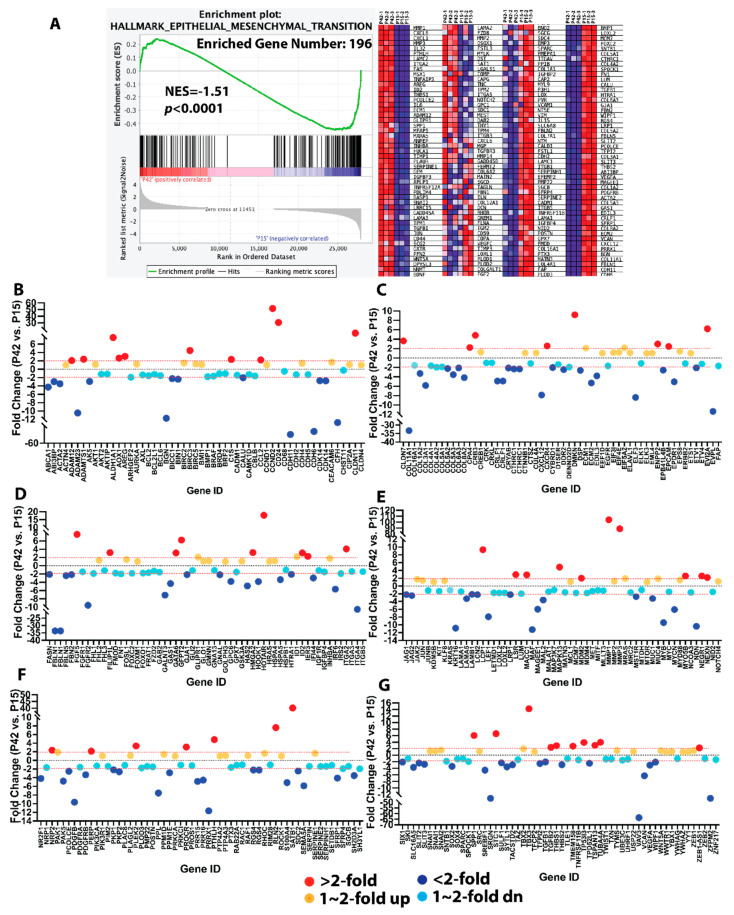
Aged LECs systematically downregulate EMT-promoting genes. (**A**) GSEA (gene set enrichment analysis) revealed that EMT is a major pathway enriched in P15 FHL124 cells with a nominal *p*-value < 0.0001, FDR = 0.037, and NES = −1.51. The left panel is the enrichment plot, and the right panel is the heatmap of 196 differentially expressed genes (DEGs) between P15 and P42 passage FHL124 cells enriched by GSEA. (**B**–**G**) DEGs analysis of 334 top-ranking EMT-promoting genes in P42 and P15 passage FHL124 cells. Gene expression is expressed as fold change between P42 and P15 passage FHL124 cells. Red dot: >2-fold upregulated; orange dot: >1 but <2-fold upregulated; dark blue dot: >2-fold downregulated; light blue dot: >1 but <2-fold downregulated.

Most surprisingly, epithelial-mesenchymal-transition (EMT) was significantly enriched in young FHL124 lens epithelial cells with a nominal *p*-value less than 0.0001 and a 3.7% FDR q-value (Figure 3A). To gain a full picture of this cellular process, we performed a differential analysis of the 334 top-ranking genes selected from pathway enrichment analysis [26,31], the pan-cancer web-based EMTome portal [32], and Ingenuity Pathway Analysis (IPA, Qiagen). These genes have been shown to positively affect or induce the EMT process. Intriguingly, aged LECs showed more than a 2-fold downregulation of 110 of these 334 genes (Figure 3B–G). P42 LECs saw mild downregulation (less than 2-fold) of 107 out of 334 genes compared with P15 (Figure 3B–G). Only 55 out of 334 genes were upregulated more than 2-fold in P42 LECs compared with P15, and 72 out of 334 genes were slightly upregulated (less than 2-fold) in P42 LECs relative to P15 (Figure 3B–G). These significantly downregulated genes included several transcription factors, such as ZFPM2 (12.75-fold dn), MAF (11.24-fold dn), LEF1(7.94-fold dn), MYB (9.50-fold dn), ELF3 (8.38-fold dn), IRF6 (5.66-fold dn), and SOX2 (3.43-fold dn). We saw that the expression of a large number of genes related to the extracellular matrix was significantly downregulated in aged LECs, including fibulin 1(FBLN1, 33.63-fold dn), biglycan (BGN, 11.70-fold dn), ADAM metallopeptidase domain 23 (ADAM23, 10.45-fold dn), extracellular matrix protein 2 (ECM2, 5.28-fold dn), type III collagen (COL3A1, 5.84-fold dn), type VIII collagen (COL8A2, 4.17-fold dn), and fibronectin (FN1, 4.64-fold dn). We also noticed that the expression of several cell adhesion molecules was significantly downregulated (more than 2-fold) in P42 LECs compared with P15, including several cadherin family genes, e.g., cadherin 11 (CDH11, 50.04-fold dn) and cadherin 6 (CDH6, 46.59-fold dn), and integrins, e.g., subunit alpha 4 (ITGA4, 10.23-fold dn) and subunit beta 5 (ITGB5, 2.12-fold dn). In addition to fibronectin and collagens, the EMT hallmark gene, alpha-smooth muscle actin (αSMA), was significantly downregulated (4-fold) in P42 passage FHL124 cells compared with P15 LECs. We also observed significant (albeit less than 2-fold) downregulation of vimentin (VIM, 1.77-fold dn, *p* < 0.05)) and tenascin C (TNC, 1.25-fold dn, *p* < 0.05). Overall, these results demonstrated that systemic EMT-promoting genes were significantly downregulated in aged LECs.

### 3.5. Lens Epithelial Cells Gradually Decrease αSMA and Fibronectin Expression in Aging

**Figure 4:**
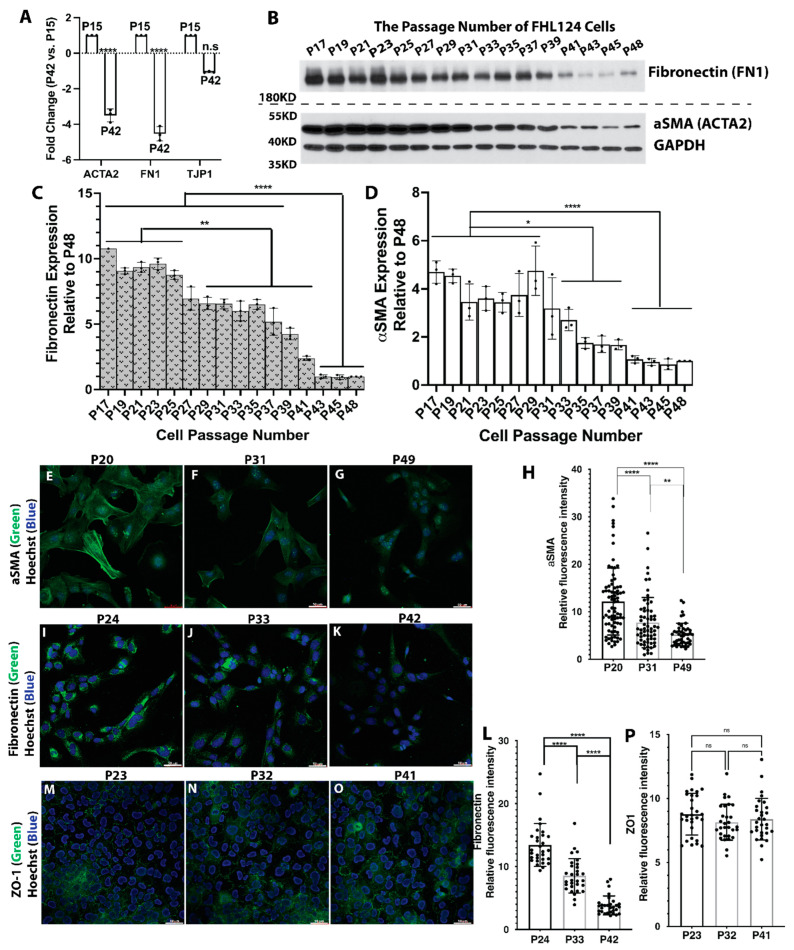
Fibronectin and αSMA protein expression gradually decreased in LECs aging. (**A**) Relative mRNA expression of fibronectin and αSMA between P42 and P15 passage FHL124 cells. Data are expressed as fold change relative to P15. (**B**) Fibronectin and αSMA protein levels from passage 17 to passage 48 FHL124 cells (n = 3). GAPDH serves as a loading control. (**C**) Semi-quantitative results of fibronectin expression from immunoblot (**B**) (n = 3). (**D**) Semi-quantitative results of αSMA expression from immunoblot (**B**) (n = 3). (**E**–**P**) immunofluorescence staining of fibronectin, αSMA, and ZO-1 protein expression in early, middle, and late passage LECs. (**E**–**G**) αSMA IF: αSMA (green), nuclei (blue) by Hoechst 33342. (**E**) Passage 20. (**F**) Passage 31. (**G**) Passage 49. (**I**–**K**) Fibronectin IF: fibronectin (green), nuclei (blue). (**I**) Passage 24. (**J**) Passage 33. (**K**) Passage 42. (**M**–**O**) ZO-1 IF: ZO-1 (green), nuclei (blue). (**M**) Passage 23. (**N**) Passage 32. (**O**) Passage 41. (**H**) Quantitative results of αSMA protein expression based on fluorescence intensity. Fluorescence intensity from 50 cells was measured. (**L**) Quantitative results of fibronectin protein expression based on fluorescence intensity. Fluorescence intensity from 50 cells was measured. (**P**) Quantitative results of ZO-1 protein expression based on fluorescence intensity. Fluorescence intensity from 50 cells was measured. Results are expressed as mean ± SD and were analyzed using one-way ANOVA with Tukey’s multiple comparisons test. Only *p* < 0.05 is considered significant. * < 0.05, ** < 0.01, *** < 0.001, **** < 0.0001; n.s, not significant.

Inspired by the surprise findings above, we decided to titrate the protein expression of several EMT hallmark genes in increased cell passages of FHL124 lens epithelial cells. αSMA and fibronectin (FN1), two classic EMT markers, and ZO-1 (TJP1), a cell tight junction marker, were tested. Figure 4A shows the relative mRNA ratios between P42 and P15 FHL124 cells. Both αSMA and fibronectin were significantly downregulated, while ZO1 was only mildly downregulated. We continuously cultured FH124 cells from passage 17 (P17) to passage 48 (P48). As shown in Figure 4B, αSMA and fibronectin protein expression gradually decreased with increased cell passages, and a significant decline of αSMA and fibronectin expression was seen around passage 31 (P31) as contrasted with passage 17 (P17) (Figure 4C,D).

Since the ZO-1 antibody we obtained was only suitable for immunocytochemistry (ICC), we also performed immunofluorescence (IF) staining of FHL124 cells at their early, middle, and late passages. Figure 4E–H (αSMA), Figure 4I–L (fibronectin), and Figure 4M–P (ZO-1) depict IF and statistical data based on the relative fluorescence intensity of αSMA, fibronectin, and ZO-1, respectively. A profound decline of immunofluorescence intensity was seen in higher passage FHL124 cells for both αSMA and fibronectin staining. There was no remarkable difference in ZO-1 expression between early, middle, and late passage cells. These results strongly support the RNAseq results, which indicate that aged lens epithelial cells suppress the EMT process.

The most altered genes from early to late passage FHL124 cells were cadherin 11 (CDH11) followed by cadherin 6 (CDH6), with each downregulated around 50-fold and 45-fold in P42 LECs compared with P15, respectively. Figure S3A shows common cadherin genes, including E-cadherin (CDH1) and N-cadherin (CDH2). The former is an indicator of epithelial cell integrity, and the latter is an indicator of cell transformation, i.e., EMT. In addition to CDH11 and CDH6, we also found several cadherin family genes that were significantly downregulated, including cadherin 3, 8, 15, 18, and 26. However, when we compared the relative intracellular mRNA levels, extremely low mRNA levels of E-cadherin were seen in both early and late passage FHL124 cells. For reference, cell tight junction gene ZO-1 expression levels were around 280-fold compared to E-cadherin (Figure S3B). CDH8, 15, 18, and 26 were also barely detectable. N-cadherin was the most dominant cadherin gene expressed in FHL124 cells, followed by CDH11 and CDH6 (Figure S3B). Interestingly, there were no remarkable changes in N-cadherin mRNA levels between P42 and P15 FHL124 cells (Figure S3A).

We confirmed the above findings at the protein level by titrating N-cadherin and CDH11 with increased cell passages. As shown in Figure S3C,D with increased cell division from passage 17 to passage 48, no remarkable N-cadherin protein expression changes were seen across 31 cell passages. In contrast, a gradual decline of CDH11 expression was seen in FHL124 cells, and a profound reduction of CDH11 protein expression was observed after passage 33 (Figure S3C,D).

### 3.6. High Passage Lens Epithelial Cells Are Less Sensitive to TGFβ2-Mediated EMT

**Figure 5:**
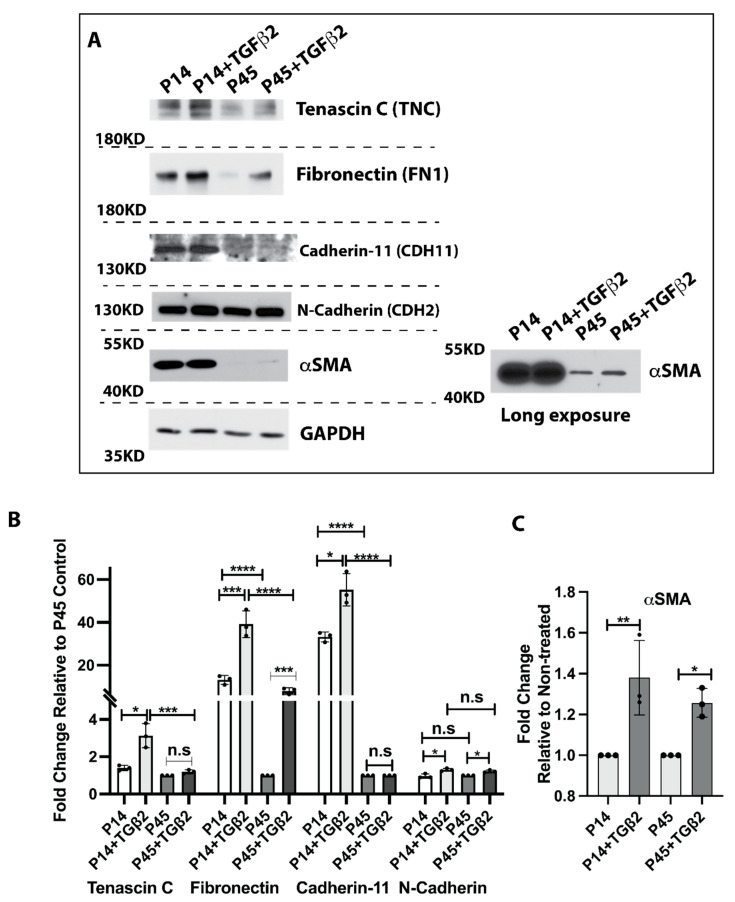
Aged LECs mitigate TGFβ2-mediated EMT. Passage 14 and 45 FHL124 cells were treated with 10 ng/mL TGFβ2 for 24 h. (**A**) Protein levels of fibronectin, cadherin 11, tenascin C, N-cadherin, and αSMA were determined by immunoblot assay of both P14 and P45 passage FHL124 cells with and without TGFβ2 treatment. A long exposure image of αSMA was included to measure αSMA expression in P45 passage cells. (**B**) Semi-quantitative results of tenascin C, fibronectin, cadherin 11, and N-cadherin protein expression. Data are expressed as fold change relative to P45 non-treated cells (control). (**C**) Semi-quantitative results of αSMA protein expression. Data are expressed as fold change relative to non-treated cells of the same passage. (**B**,**C**) Results are expressed as mean ± SD and were analyzed using one-way ANOVA with Tukey’s multiple comparisons test. Only *p* < 0.05 is considered significant. * < 0.05, ** < 0.01, *** < 0.001, **** < 0.0001; n.s, not significant.

TGFβ is one of the most potent EMT inducers, and TGFβ-induced EMT is a well-recognized mechanism in the PCO initiation and progression [15,33]. To test young and aged LECs’ response to TGF-mediated EMT signaling, we treated young (passage 14, P14) and aged (passage 45, P45) FHL124 cells with 10 ng/mL TGFβ2 for 24 h. As shown in Figure 5A–C, without TGFβ2 stimulation, the baseline expression of cadherin 11 (CDH11), αSMA, and fibronectin was significantly lower in passage 45 FHL124 cells compared with passage 14. There was a mild decrease in tenascin C (TNC) expression, and no noticeable expression changes were seen in N-cadherin (CDH2) when comparing P45 with P14 FHL124 cells. After 24 h TGFβ2 treatment, significantly increased tenascin C (TNC), cadherin 11 (CDH11), αSMA, fibronectin, and N-cadherin (CDH2) were seen in P14 FHL124 cells compared with non-treated P14 cells. Although we found that fibronectin and N-cadherin expression was significantly higher in TGFβ2 treated P45 cells compared with non-treated P45 cells, we did not see a remarkable increase of tenascin C and cadherin 11 expression in TGFβ2 treated P45 cells compared with non-treated P45 cells (Figure 5A–C). αSMA expression was only detectable after a long time of exposure in P45 FHL124 cells with and without TGFβ2 treatment (Figure 5A). Importantly, the levels of these EMT markers were markedly decreased in TGFβ2 treated P45 compared with TGFβ2 treated P14 FHL124 cells (Figure 5A–C). Overall, these results demonstrate that aged lens epithelial cells mitigate TGFβ2-induced EMT signaling.

### 3.7. Aged Mice Alleviate Lens Epithelial Cell EMT Signaling after Cataract Surgery

**Figure 6:**
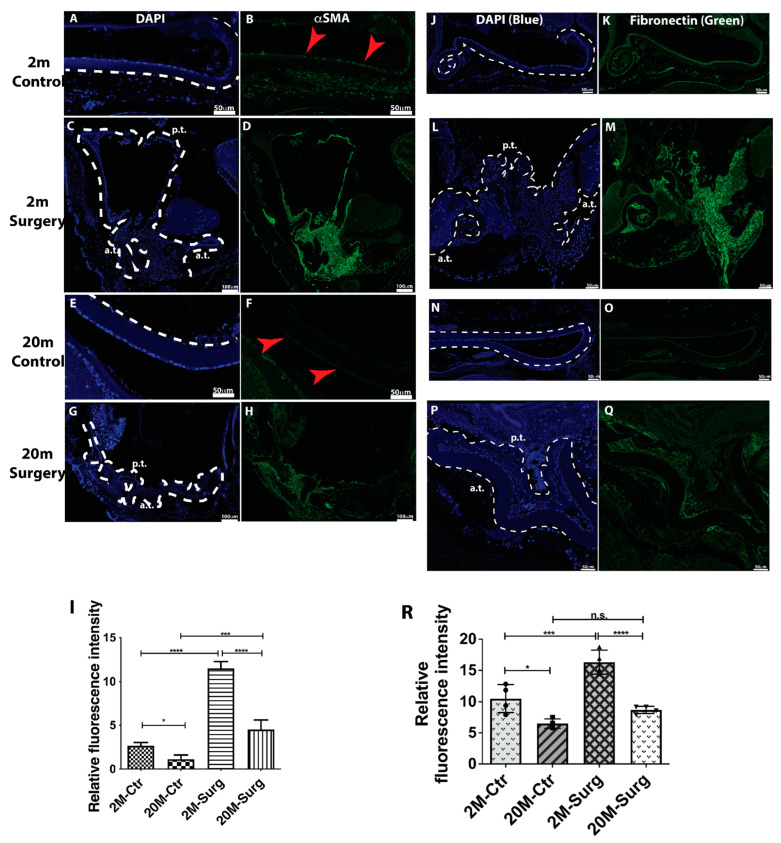
αSMA and fibronectin protein expression in 2- and 20-month-old mouse lens capsules after cataract surgery. (**A**,**C**,**E**,**G**) Cell nucleus staining by DAPI is shown in blue. (**B**,**D**,**F**,**H**) αSMA antibody staining is shown in green. (**A**) Two-month control, DAPI. (**B**) Two-month control, αSMA. (**C**) Two-month surgery, DAPI. (**D**) Two-month surgery, αSMA. (**E**) Twenty-month control, DAPI. (**F**) Twenty-month control, αSMA. (**G**) Twenty-month surgery, DAPI. (**H**) Twenty-month surgery, αSMA. (**I**) Quantitative results of αSMA protein expression based on fluorescence intensity (n = 4). (**J**,**L**,**N**,**P**) Cell nucleus stain by DAPI is shown in blue. (**K**,**M**,**O**,**Q**) Fibronectin antibody stain is shown in green. (**J**) Two-month control, DAPI. (**K**) Two-month control, fibronectin. (**L**) Two-month surgery, DAPI. (**M**) Two-month surgery, fibronectin. (N) Twenty-month control, DAPI. (**O**) Twenty- month control, fibronectin. (**P**) Twenty-month surgery, DAPI. (**Q**) Twenty-month surgery, fibronectin. (**R**) Quantitative results of fibronectin protein expression based on fluorescence intensity (n = 4). Results are expressed as mean ± SD and were analyzed using one-way ANOVA with Tukey’s multiple comparisons test. Only *p* < 0.05 is considered significant. * < 0.05, ** < 0.01, *** < 0.001, **** < 0.0001; n.s., not significant.

The substantial evidence above was based on the cellular aging model study, and an obvious question is whether this is also happening in the *in vivo* situation. To test this, we used the mouse cataract surgery model. We performed mouse cataract surgery on 2- and 20-month-old mice and determined the EMT signaling five days after surgery. Mice were immediately euthanized and processed after cataract surgery to serve as a control. As shown in Figure 6A,B (2 mos) and Figure 6E,F (20 mos) of the control group, a stronger αSMA staining signal was seen in 2-month-old mouse lens epithelium compared with 20- month-old mouse lens epithelium. Semi-quantitative data indicated that epithelium αSMA levels were significantly higher in 2-month-old mice than 20-month-old mice (Figure 6I). There was a drastic increase in αSMA in 2-month-old lens capsules five days after surgery. αSMA-positive cells were not only seen clustering around the anterior capsule but also along with the posterior capsule (Figure 6C,D). We did observe αSMA-positive staining cells 5 days after cataract surgery in 20-month-old mice, but it was markedly lower than in two-month-old surgery mice (Figure 6G–I). Similar results were seen in fibronectin immunostaining. Baseline fibronectin was considerably higher in 2-month-old mouse epithelium than 20-month-old mouse epithelium (Figure 6J,K (2 mos) and Figure 6N,O (20 mos), Figure 6R), and cataract surgery substantially increased fibronectin expression in both 2-month-old and 20-month-old mice. However, levels of fibronectin in 2-month-old surgery mice were significantly higher than in 20-month-old surgery mice (Figure 6L,M (2 mos), Figure 6P,Q (20 mos), Figure 6R). Tenascin C was barely detectable in control lens epithelium from both 2- and 20-month-old mice, and a remarkable elevation of tenascin C expression was seen five days after cataract surgery (Figure S4). Significantly higher levels of tenascin C were seen in 2-month-old lens capsules than 20-month-old lens capsules (Figure S4). These results strongly suggest that aged lens epithelial cells suppress EMT signaling in *in vivo* conditions mimicking cataract surgical procedures.

## 4. Discussion

A profound difference in the PCO incidences between young and aged cataract surgery patients implies that there must be some fundamental differences between young and old lens epithelial cells or the surrounding environment. Consequently, these differences provide an excellent point of entry to studying mechanisms of PCO formation. The present study focuses on the lens epithelial cells’ age-related changes by conducting a comprehensive comparative transcriptomic analysis between young and aged LECs. We found that aged LECs not only decelerated rates of cell proliferation but also suppressed LEC EMT through systemically downregulating EMT-promoting genes. These findings were confirmed in the in vitro TGFβ2-mediated EMT signaling and the *in vivo* mouse cataract surgery model studies. 

There was some debate regarding which aging model we should choose for this study, i.e., lens epithelium isolated from collected human lenses at young and old age versus an in vitro cellular aging model. We believed that LECs harvested from intact human lenses might not sufficiently reflect the actual cell growth conditions after cataract surgery. LECs within an entire lens are enclosed by the lens capsule, and the LECs’ apical side faces lens fibers, a relatively sealed environment. On the contrary, the apical side of residual LECs on the lens capsule after cataract surgery faces a continuously circulating aqueous humor, a relatively open environment. After cataract surgery, LECs on the lens capsule from either young or aged patients will first adapt to an opened growth condition closer to the *in vitro* cell culture scenario. However, we did see very similar age-related gene expression changes between our cell culture model and the recent aging mouse epithelium transcriptome study [25], such as upregulated immune response and cellular stress responses. Collecting LECs from cataract surgery donors would be more problematic because donors’ cataract surgeries were often done years ago, and LECs may already be situated in various PCO stages. FHL124 cells are spontaneously grown out of the lens epithelial cell line without any additional immortalization. It is an ideal cell suitable for a cellular aging model to study LEC aging. In fact, even several immortalized mouse lens epithelial cell lines were found to have high homology to the lens epithelium [34]. However, the in vitro cultured LECs may significantly alter some gene expressions compared with lens epithelium since the 5% FBS used in the culture medium is different from the physiological condition. One key example is that FHL124 cells have higher αSMA endogenous expression levels than human epithelium, but we expect their age-related behavior and adaptation would be similar. Importantly, we have demonstrated that the FHL124 cellular aging model closely resembles the *in vivo* situation [21]. Furthermore, in vitro cellular aging culture models are widely used and well-received in aging studies [35,36,37]. In the present study, conclusions drawn from the in vitro FHL124 cellular aging study agree with the *in vivo* mouse cataract surgery study.

The present study yielded well-anticipated results that aged LECs decelerate cell proliferation rates. Pathway and gene enrichment analysis indicate that either cell survival or cell proliferation promoting pathways are enriched in young LECs, such as the hedgehog signaling [38], the UPR pathway [39], and the MTORC1 signaling [40]. In contrast, cell apoptosis and cell stress response pathways, such as ROS and oxidative phosphorylation pathways [41], are enriched in aged LECs. For this study, we used FHL124 cells at least five passages ahead of the complete cell senescence [21], and this was primarily confirmed based on cell doubling times. However, although most senescent genes remained unchanged, we did observe some cell senescent genes that were up- or downregulated to promote cell senescence. These changes suggest that aged LECs at passage 42 or so are in the transition stage of acquiring a senescence-like phenotype. In fact, deregulation in immune response, upregulated cytokines, and immune modulators in aged LECs likely result from senescence-associated secretory phenotype (SASP), a well-known cell senescence phenotype [42]. Declined cell proliferation in the lens epithelium is also supported by work from both human and rodent studies. Li et al. [43] found that the proliferative capacity of LECs in old mice was decreased significantly compared with LECs from young mice. Using the human capsular bag culture model, Wormstone et al. [19] found much higher LEC proliferation rates on lens capsules from donors aged <40 years compared with >60 years. In a follow-up study, Dawes et al. [44] demonstrated that the intracellular signaling might be responsible for high cell proliferation in the young lens capsule rather than secreted cytokines in the medium. Our present study supports the notion that systemic regulatory pathways in young and aged LECs determine the rates of cell division. However, the impact of cytokines from the medium and cell growth environment in LEC transformation, i.e., EMT, remains to be clarified.

The present study also yielded a surprise finding that aged LECs systemically downregulated signaling pathways to suppress EMT. An EMT suppressive behavior in aged LECs is contrary to epithelial or endothelial cells from many tissues that have been shown to acquire EMT characteristics during aging, which adversely cause organ fibrosis via altering epithelial or endothelial cell phenotypic properties and depositing extracellular matrix (ECM). This includes kidney, cardiac, and idiopathic pulmonary fibrosis [45,46,47,48,49]. We found that hundreds of EMT-promoting genes, including several key EMT markers, such as fibronectin and αSMA, were significantly downregulated. Importantly, we demonstrated that FHL124 cells mitigate TGFβ2-mediated EMT signaling at their higher passages. Furthermore, we have explicitly confirmed our in vitro findings through *in vivo* mouse cataract surgery studies. Several EMT hallmark genes, including fibronectin, αSMA, and tenascin C, are markedly increased in mouse lens capsules at 2 months compared with 20 months after cataract surgery. In addition, substantially decreased baseline fibronectin and αSMA expression from 2-month-old to 20-month-old mouse lens epithelium agrees with our in vitro cellular aging model study. We also had another surprise finding that a well-known cell adhesion molecule, E-cadherin (CDH1), is barely expressed in FHL124 cells from RNAseq. We could not detect E-cadherin protein with a widely used commercial antibody. N-cadherin (CDH2), on the other hand, is abundantly expressed in FHL124 cells but does not alter with age and has only a mild TGFβ2-mediated response. Interestingly, cadherin 11 (CDH11), an emerging gene marker and a top-ranked EMT gene [32,50,51,52], is moderately expressed in young FHL124 cells and demonstrates an age-related decline in FHL124 cells. Along with the positive TGFβ2-mediated response, we believe CDH11 may be used as a sensitive EMT marker for LECs. Unfortunately, several commercially available CDH11 antibodies we tested in this study only work for the Western blot, and antibodies capable of immunohistology are urgently needed.

The present study suggests that not only do rapid proliferative LECs at a young age lead to increased PCO incidence, but pro-EMT characteristics of young LECs may also inevitably accelerate PCO progression. Conversely, slow LEC division and systematic EMT suppression in aged lenses collaboratively slow PCO initiation and progression. Downregulation of a wide scale of pro-EMT genes, from young to aged LECs, also implies a significant challenge in preventing PCO development, especially in infant and children cataract patients. This is most likely because complex and multifactorial pathways are involved in the pathogenesis of PCO formation. Future studies are needed to dissect these pathways and multi-pathway crosstalk, which will significantly increase the identification of therapeutic targets. For that purpose, we believe the database established in the present study may serve as a valuable resource. However, we are aware that this study focuses solely on the LECs’ gene expression shift during aging. The microenvironment after cataract surgery and its impact on PCO initiation and progression remain to be elucidated.

Taken all together, our present study implicates that the discrepancy of rates of PCO incidence between young and aged cataract surgery patients results at least from both cellular proliferation capacity and pro-EMT characteristics. Unravelling these players will help us gain a significant insight that can assist in identifying therapeutic targets.

## Data Availability

RNAseq data are deposited in the Gene Expression Omnibus (GEO) database under accession number GSE184422. The authors will provide a detailed description of methods and original data upon request.

## Supplementary Materials

The following supporting information can be downloaded at: https://www.mdpi.com/article/10.3390/cells11132001/s1, Table S1. Common and distinct genes in human FHL124 LECs vs. three mouse lens epithelium in their top 500 most expressed genes; Figure S1. RNAseq data analysis summary. (A) volcano plot comparing P42 to Pl 5 FHL 124 cells. (B) Principal component analysis (PCA); Figure S2. DEGs analysis of cell senescence genes. Figure S3. Cadherin 11 (CDH11) is an EMT marker for LECs. (A) RNAseq data of cell adhesion cadherin family molecules. Data are expressed as fold change of P42 vs. Pl 5 passage FHL 124 cells. Dark blue dot: >2-fold downregulated; light blue dot: >1 but <2-fold downregulated; orange dot: >1 but <2-fold upregulated. (B) The relative mRNA levels of selected cadherin genes are measured by normalized mean count of transcripts per million (TPM). ZO-1 (TJP1) serves as a reference cell junction gene. (C) N-cadherin and CDH11 protein expression determined by immunoblot. GAPDH serves as a loading control. (D) Semi-quantitative results of N-cadherin and CDH11 protein levels in FHL 124 cells at various passages. Results are expressed as mean ± SD and were analyzed using one-way ANOVA with Tu key’s multiple comparisons test. Only *p* < 0.05 is considered significant. * *p* < 0.05, ** *p* < 0.01, *** *p* < 0.001, **** *p* < 0.0001, n.s, not significant; Figure S4. Tenascin C protein expression in 2- and 20-months old mouse lens capsules after cataract surgery. (A,C,E,G) cell nucleus staining by DAPI is shown in blue. (B,D,F,H) Tenascin C antibody staining is shown in green. (A) 2 months control, DAPI.(B) 2 months control, Tenascin C. (C) 2 months surgery, DAPI. (D) 2 months surgery, Tenascin C. (E) 20 months control, DAPI. (F) 20 months control, Tenascin C. (G) 20 months surgery, DAPI. (H) 20 months surgery, Tenascin C. (I) Quantitative results of Tenascin C protein expression based on fluorescence intensity (n = 4). Results are expressed as mean± SD and were analyzed using one-way ANOVA with Tu key’s multiple comparisons test. Only *p* < 0.05 is considered signi cant. * *p* < 0.05, ** *p* < 0.01, *** *p* < 0.001, **** *p* < 0.0001, ns. not significant.
