# Supplementary material for: Aged Lens Epithelial Cells Suppress Proliferation and Epithelial–Mesenchymal Transition-Relevance for Posterior Capsule Opacification
Source: Cells. 2022 Jun 22;11(13):2001. doi: 10.3390/cells11132001 (PMC9265589; doi:10.3390/cells11132001)
Supplement: Supplementary file 1 [file cells-11-02001-s001.zip › cells-1726348-supplementary.pdf]

Table S1. Common and distinct genes in human FHL124 LECs vs. three mouse lens epithelium in their top 500 most expressed genes

**Common Genes List**			**Distinct Genes List**		
179 of 254 shared genes in 3 independently reported mouse lens epithelium are also FHL124 top 500 expressed genes			*75 of 254 shared genes in 3 independently reported mouse lens epithelium are not in FHL124 top 500 expressed genes*		
ACTB	MORF4L1	RPL38	AKR1A1	CST3	NID1
ACTG1	MRFAP1	RPL39	ALDH1A1	CTSD	NREP
ALDOA	MT-ATP6	RPL4	ARGLU1	CTSL	ODC1
ANXA1	MT-ATP8	RPL41	ARHGDIA	DKK3	PGK1
APP	MT-CO1	RPL6	ASS1	EEF1B2	PMP22
ARF1	MT-CO2	RPL7	ATP1B3	FOXE3	R3HDML
ATP1A1	MT-CO3	RPL7A	ATP6V0C	GJA3	RPL23A
ATP5A1	MT-ND1	RPL8	BC1	GJA8	RPL36
ATP5B	MT-ND2	RPL9	BTG1	GNG5	RPL36A
BSG	MT-ND3	RPLP0	CADM1	GRIFIN	RPS25
BTF3	MT-ND4	RPLP1	CD81	GSTM1	RPS26
CALM2	MT-ND4L	RPS11	CDH1	H3F3A	RPS28
CALM3	MT-ND5	RPS12	CLU	HIF1A	SELENOW
CALR	MT-ND6	RPS13	COX4I1	IGFBP7	SFRP1
CANX	MT-RNR1	RPS14	COX6C	ITM2B	SKP1A
CCT2	MT-RNR2	RPS15	COX8A	LIX1	SLC25A4
CCT5	NACA	RPS15A	CPE	MAGED1	SLC2A1
CD63	NPM1	RPS16	CRYAA	MGARP	SLC7A8
CDH2	OAZ1	RPS17	CRYAB	MIF	TMEM59
CFL1	P4HB	RPS18	CRYBA1	MIP	TUBB5
CHCHD2	PABPC1	RPS19	CRYBA2	MRFAP1	UBB
CNN3	PDIA3	RPS2	CRYBA4	MT-CYTB	UQCRH
COL4A1	PEBP1	RPS20	CRYBB1	NAP1L1	VIT
COL4A2	PFN1	RPS21	CRYBB3	NDUFA1	SOD1
COTL1	PGAM1	RPS23	CRYGS	NEDD4	SPON1
CRIM1	PHGDH	RPS24			
DDOST	PKM	RPS27A			
DDX5	PPIA	RPS29			
DSTN	PPIB	RPS3			
EEF1A1	PSAP	RPS3A			
EEF1G	PTMA	RPS4X			
EEF2	PTMS	RPS5			
EIF1	PXDN	RPS6			
EIF4A1	RAC1	RPS7P1			
EIF4B	RCN1	RPS8			
EIF4G2	RPL10	RPS9			
EIF5A	RPL11	RPSA			
ENO1	RPL13	SCD			
EZR	RPL13A	SERF2			
FSTL1	RPL14	SLC25A3			
FTH1	RPL18	SLC25A5			
FTL	RPL18A	SLC38A2			
GAPDH	RPL19	SLC3A2			
GJA1	RPL22	SLC7A5			
GNAS	RPL23	SPARC			
H3F3B	RPL24	SSR3			
HMGN2	RPL26	STMN1			
HNRNPA1	RPL27	TKT			
HNRNPA2B1	RPL27A	TMBIM6			
HNRNPU	RPL28	TMSB10			
HSP90AA1	RPL29	TMSB4X			
HSP90AB1	RPL3	TPT1			
HSP90B1	RPL30	TUBA1A			
HSPA5	RPL31	TUBA1B			
HSPA8	RPL32	UBB			
HSPG2	RPL34	VIM			
ITGB1	RPL35	YBX1			
LDHA	RPL35A	YWHAE			
MALAT1	RPL37	YWHAQ			
	RPL37A	YWHAZ			

Figure S1

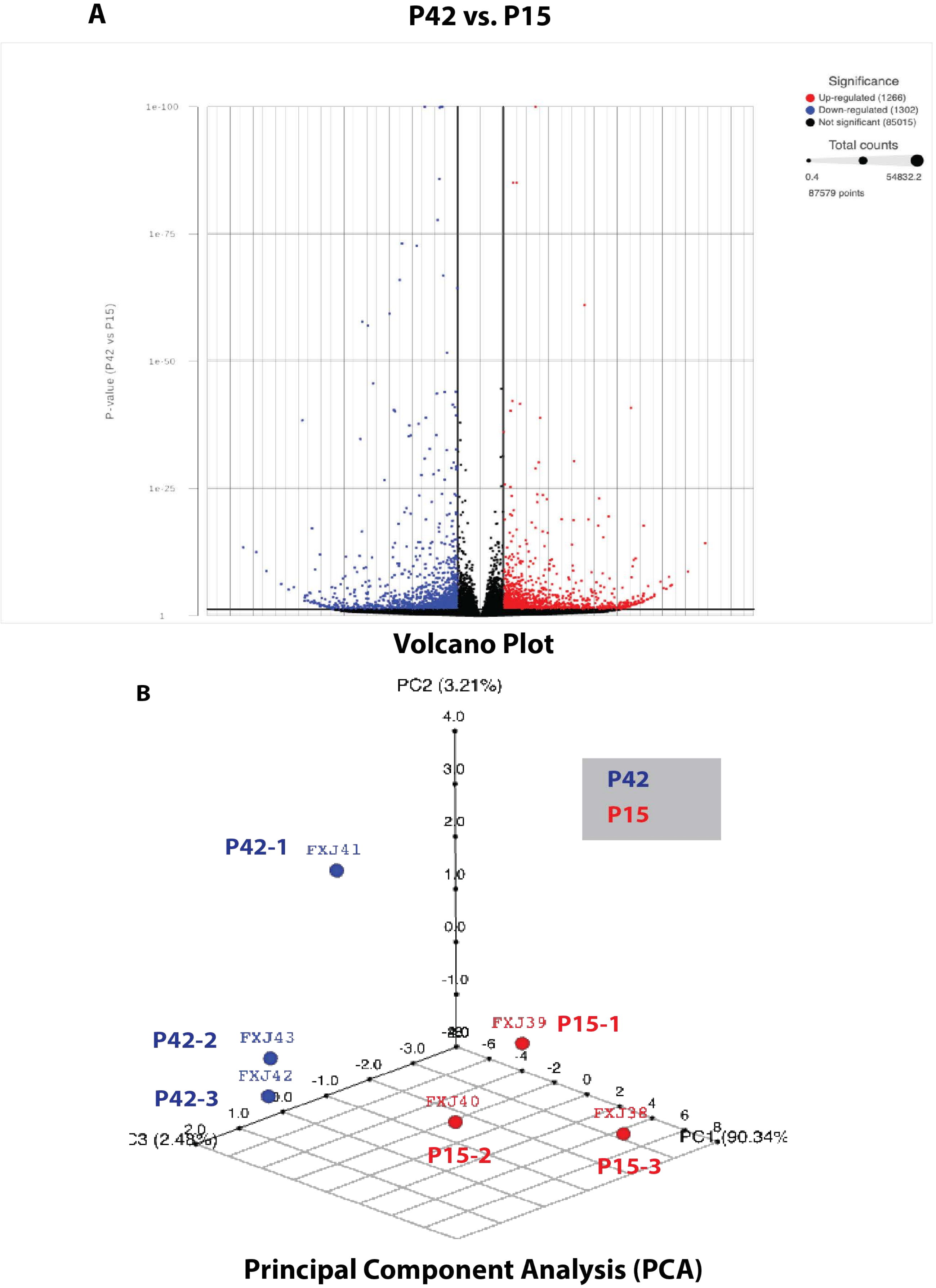

Figure S1. RNAseq data analysis summary. (A) volcano plot comparing P42 to P15 FHL124 cells. (B) Principal component analysis (PCA).

Figure S2

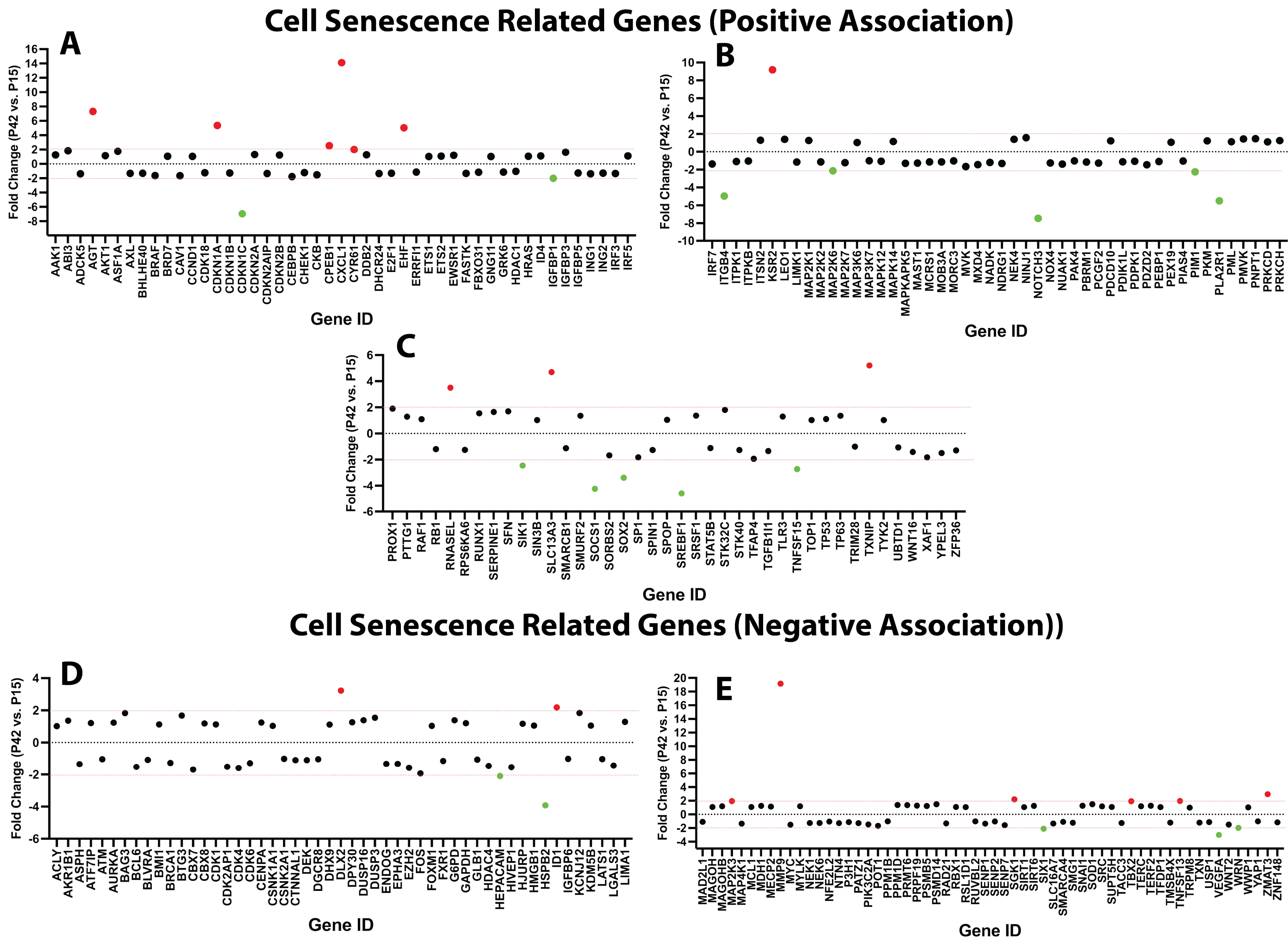

Figure S2. DEGs analysis of cell senescence related genes. (A, B, C) DEGs positively associated with cell senescence. (D, E) DEGs negatively associated with cell senescence. Data are expressed in fold change between P42 and P15 passage FHL124 cells. Red dot: >2-fold upregulated; Green dot: >2-fold downregulated; Black dot: >1 but <2-fold up-or down-regulated.

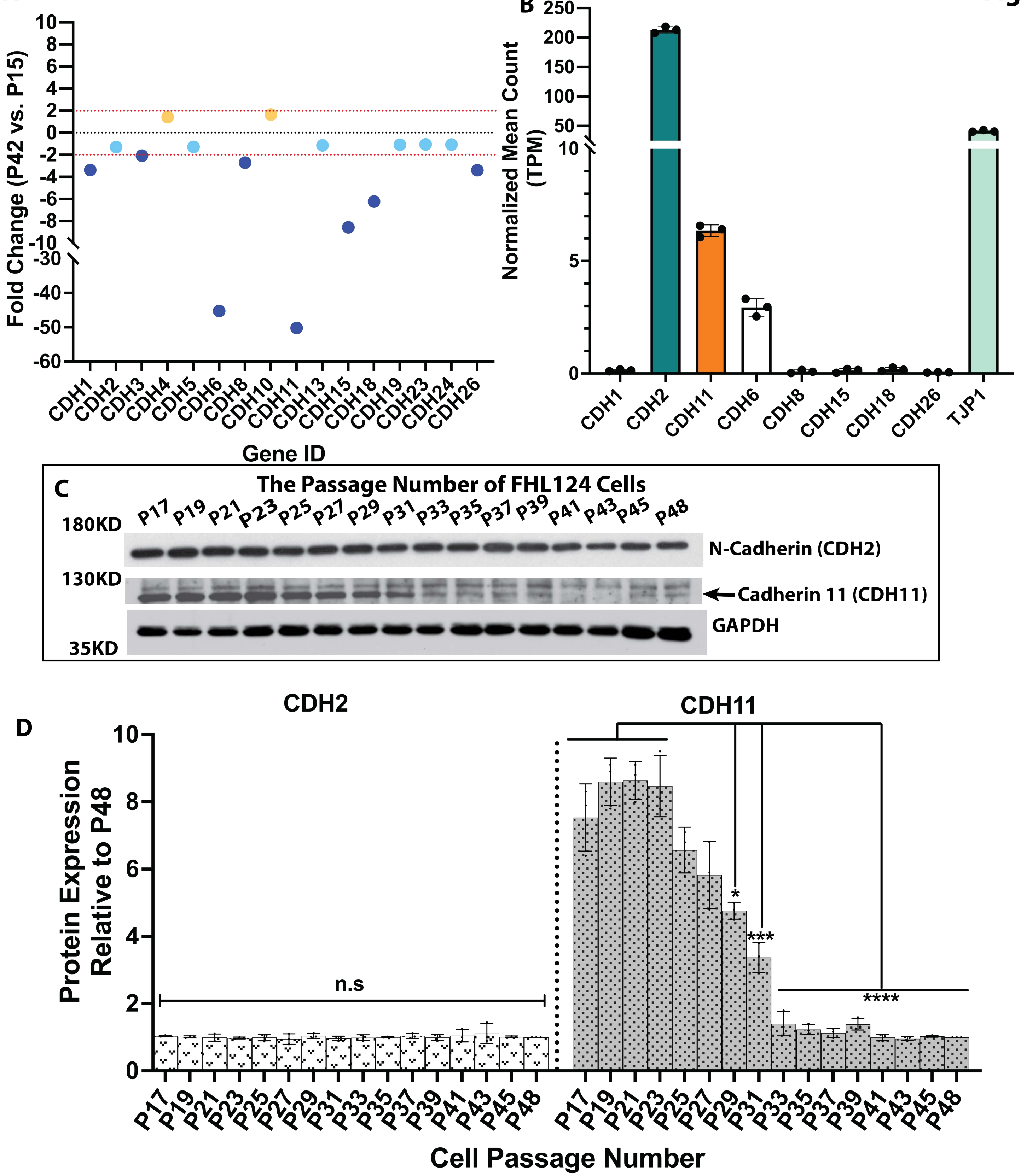

Figure S3. Cadherin 11 (CDH11) is an EMT marker for LECs. (A) RNAseq data of cell adhesion cadherin family molecules. Data are expressed as fold change of P42 vs. P15 passage FHL124 cells. Dark blue dot: >2-fold downregulated; light blue dot: >1 but <2-fold downregulated; orange dot: >1 but <2-fold upregulated. (B) The relative mRNA levels of selected cadherin genes are measured by normalized mean count of transcripts per million (TPM). ZO-1 (TJP1) serves as a reference cell junction gene. (C) N-cadherin and CDH11 protein expression determined by immunoblot. GAPDH serves as a loading control. (D) Semi-quantitative results of N-cadherin and CDH11 protein levels in FHL124 cells at various passages. Results are expressed as mean ± SD and were analyzed using one-way ANOVA with Tukey's multiple comparisons test. Only $p<0.05$ is considered significant. *$p<0.05$, **$p<0.01$, ***$p<0.001$, ****$p<0.0001$, n.s, not significant.

Figure S4

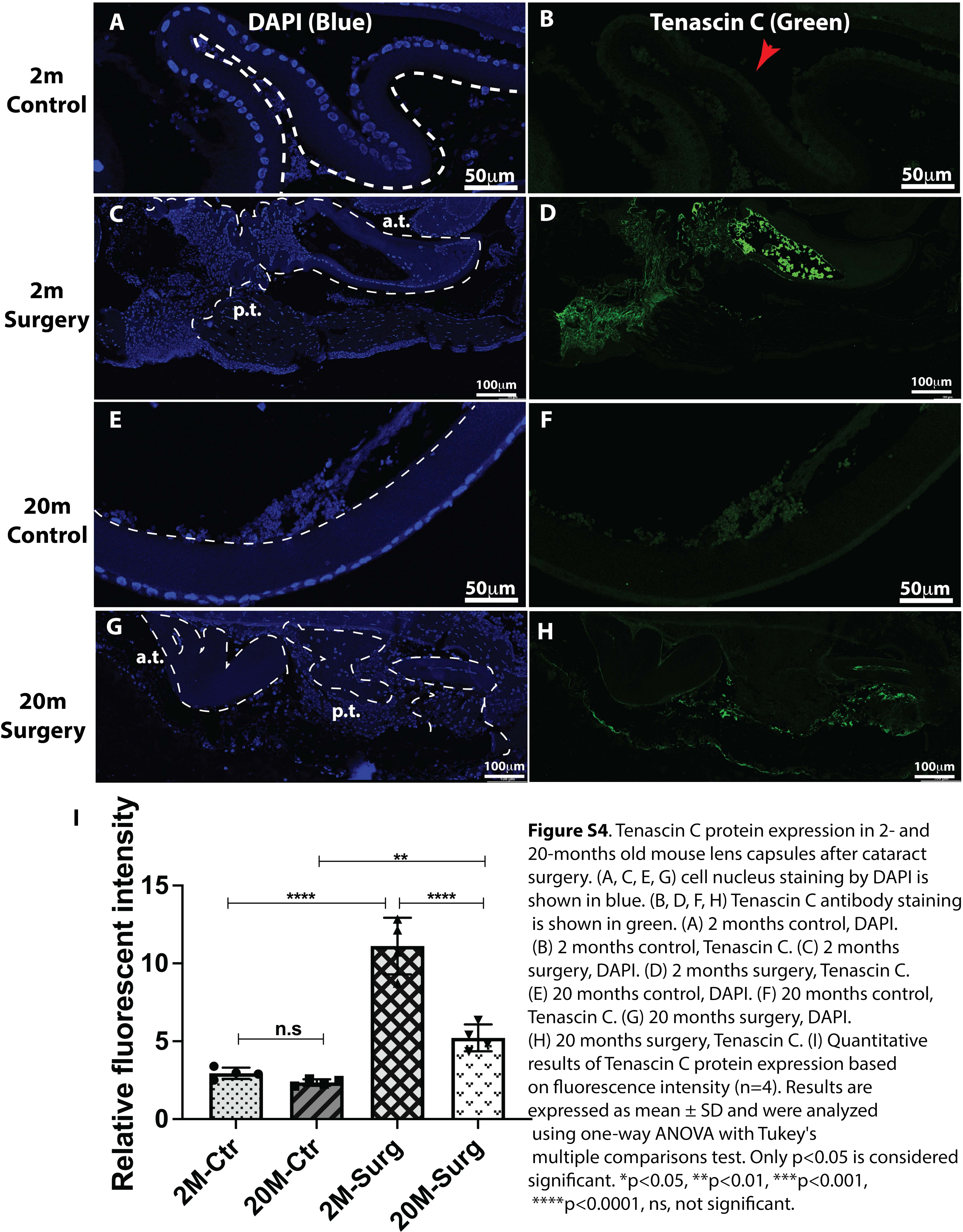

Figure S4. Tenascin C protein expression in 2- and 20-months old mouse lens capsules after cataract surgery. (A, C, E, G) cell nucleus staining by DAPI is shown in blue. (B, D, F, H) Tenascin C antibody staining is shown in green. (A) 2 months control, DAPI. (B) 2 months control, Tenascin C. (C) 2 months surgery, DAPI. (D) 2 months surgery, Tenascin C. (E) 20 months control, DAPI. (F) 20 months control, Tenascin C. (G) 20 months surgery, DAPI. (H) 20 months surgery, Tenascin C. (I) Quantitative results of Tenascin C protein expression based on fluorescence intensity (n=4). Results are expressed as mean ± SD and were analyzed using one-way ANOVA with Tukey's multiple comparisons test. Only $p<0.05$ is considered significant. *$p<0.05$, **$p<0.01$, ***$p<0.001$, ****$p<0.0001$, ns, not significant.
